# Quantitative Mass Spectrometry-Based Proteomic Profiling for Precision Medicine in Prostate Cancer

**DOI:** 10.3389/fonc.2017.00267

**Published:** 2017-11-07

**Authors:** Amilcar Flores-Morales, Diego Iglesias-Gato

**Affiliations:** ^1^Faculty of Health and Medical Sciences, Department of Drug Design and Pharmacology, University of Copenhagen, Copenhagen, Denmark; ^2^Danish Cancer Society Research Center, Danish Cancer Society, Copenhagen, Denmark

**Keywords:** mass spectrometry, prostate cancer proteomics, proteome, FFPE, biofluid proteomics, prognostic biomarker, diagnostic biomarker

## Abstract

Prostate cancer (PCa) is one of the most frequently diagnosed cancer among men in the western societies. Many PCa patients bear tumors that will not threat their lives if left untreated or if treatment is delayed. Our inability for early identification of these patients has resulted in massive overtreatment. Therefore, there is a great need of finding biomarkers for patient stratification according to prognostic risk; as well as there is a need for novel targets that can allow the development of effective treatments for patients that progress to castration-resistant PCa. Most biomarkers in cancer are proteins, including the widely-used prostate-specific antigen (PSA). Recent developments in mass spectrometry allow the identification and quantification of thousands of proteins and posttranslational modifications from small amounts of biological material, including formalin-fixed paraffin-embedded tissues, and biological fluids. Novel diagnostic and prognostic biomarkers have been identified in tissue, blood, urine, and seminal plasma of PCa patients, and new insights in the ethology and progression of this disease have been achieved using this technology. In this review, we summarize these findings and discuss the potential of this technology to pave the way toward the clinical implementation of precision medicine in PCa.

## Introduction

Prostate cancer (PCa) leads the statistics in cancer diagnosis and cancer-related death among men in the western societies (1). Current therapies, which are based on radiation or surgery for prostate-confined tumors and on androgen-deprivation therapy (ADT) for locally advanced or metastatic presentations have demonstrated to be very effective in the management of the disease. Unfortunately, the positive effects of these therapies are only temporary and most patients relapse after ADT into the so-called castration-resistant prostate cancer (CRPC), against which no curative therapy exists (2).

Overtreatment is a major concern in the management of PCa. Due to the slow progressing nature of PCa and the advanced age of the patient population, it is estimated that many of the surgically treated patients would not die of the disease or experience major morbidities if therapeutic action was not taken (3). However, our inability to distinguish between indolent PCa tumors and those which are life threatening has led to the problem of overtreatment (3). Thus, two current major areas of research in the PCa field are (i) the identification of biomarkers that can accurately predict the virulence of a prostate localized tumors and (ii) the development of effective treatments against lethal CRPC. It has now become clear that the application of state-of-the-art mass spectrometry-based proteomics to PCa research can contribute to address these and other questions related the clinical management of PCa (Table 1).

**Table 1:** Open questions in PCa research susceptible to be addressed by applying mass spectrometry-based proteomics to a range of biological samples.

Source		PCa clinical application	PCa biology
Biopsies	Diagnostic Active surveillance	Prognostic markers	Multifocality
Radical prostatectomies	Non-malignant tissue Tumor Reactive stroma	Prognostic markers (4–6) Therapeutic options (5, 7) Marker of therapeutic response (ADT; Chemotherapy, etc.)	Multifocality Transition normal to tumor (6, 8, 9) Histological subtypes (GS, Adeno.; NE) (6, 10) Molecular subtypes (T:E, SPOP, etc.) (11) Reactive stroma (12)
Metastasis	Proximal metastasis (lymph node) Distant metastasis (bone, soft tissue)	Prognostic markers Therapeutic options Marker of therapeutic response (ADT; chemotherapy, etc.)	Metastatic niche (13) Transition localized to metastasis (13) NE CRPC Molecular subtypes (T:E, SPOP, etc.) Disease relapse
Body fluids	Blood and blood fractions (CTCs, exosomes, etc.) Urine and urine fractions (exosomes, etc.) Seminal plasma and seminal plasma fractions (exosomes, etc.)	Diagnosis markers (14–19) Prognostic markers (15, 20–24) Marker of therapeutic response (ADT; chemotherapy, etc.)	PCa stage secretome (14, 16–18, 20, 21, 25, 26). Histological subtypes (15) secretome (GS, Adeno.; NE). Molecular subtypes secretome (T:E, SPOP, etc.) (27) Treatment influenced secretome (ADT; Chemotherapy, etc.)

*Biomarkers description recently reviewed by Pin et al. (28), Di Meo et al. (29), and Tanase et al. (30)*.

*PCa, prostate cancer; ADT, androgen-deprivation therapy; CRPC, castration-resistant prostate cancer; NE, neuroendocrine; GS, Gleason Score; CTC, circulating tumor cell; T:E, TMPRSS2:ERG; Adeno, adenocarcinoma*.

**Figure 1:**
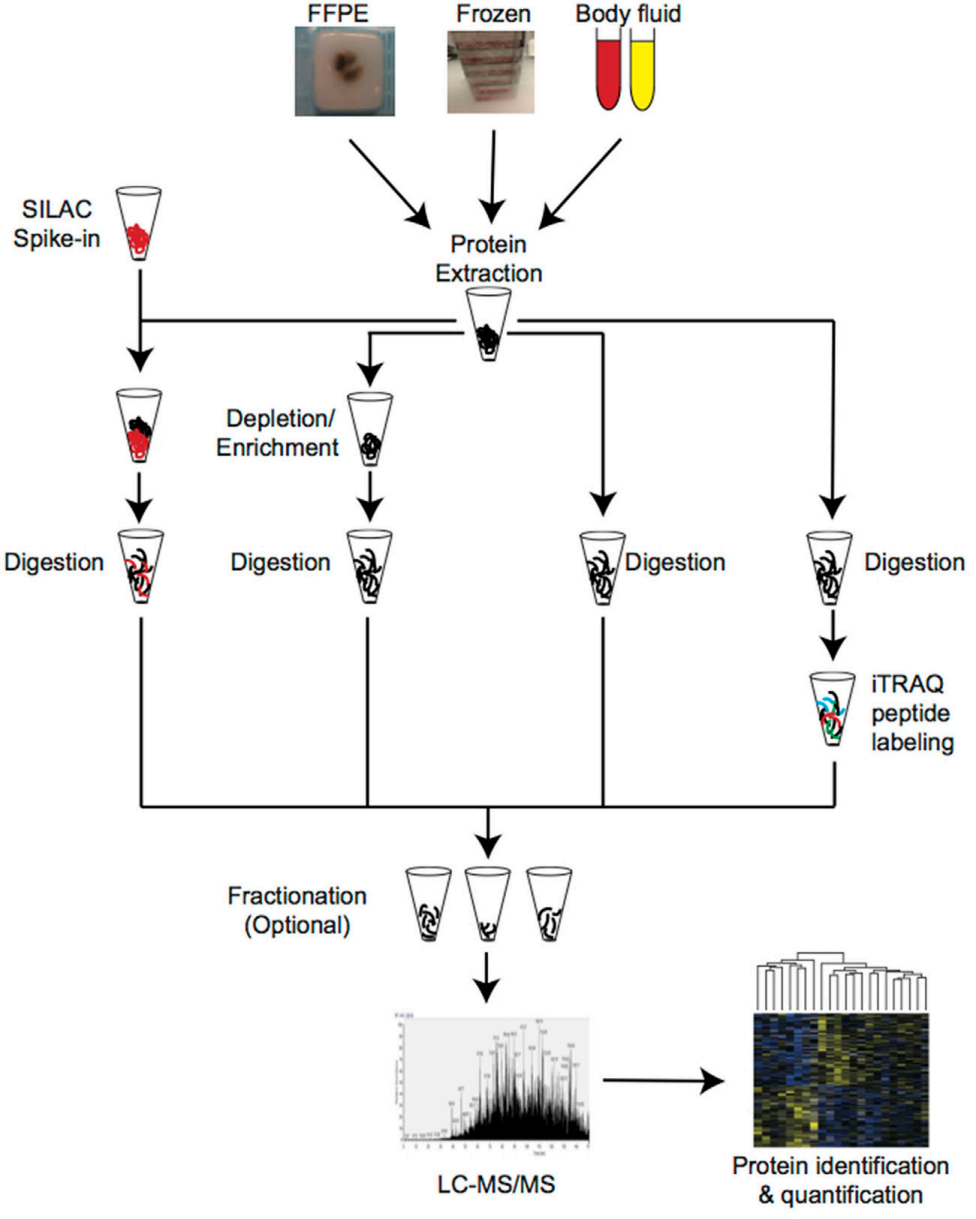
Schematic representation of the commonly used approaches for mass spectrometry-based proteomic profiling in clinical cancer research.

Proteins are the effectors of most cellular reactions and constitute the cellular targets for a majority of therapeutic drugs. Over the years, many protein biomarkers and potential drug targets have been identified using mass spectrometry techniques [recently reviewed in Ref (28–30).]. This pioneer studies used proteomic methodologies with limited capacity to achieve great depth and unfortunately most of these markers have not been further validated or implemented in the clinical setting. In-depth quantitative proteomic profiling, long-time hampered by the complexity of the proteome and the lack of appropriate technology, is now possible from both frozen and formalin-fixed-paraffin-embedded (FFPE) tissues as well as from body fluids (Figure 1) (6, 31–36). The use of mass spectrometry-based proteomic profiling to well-characterized cohorts of PCa patients with detailed clinical information have the potential to provide effective stratification of PCa patients as well as targets for novel treatments (37). In this review, we aim to summarize the achievements regarding the use of mass spectrometry-based proteomics for large-scale profiling of PCa and discuss the potential use of this technology for the identification of diagnostic and prognostic biomarkers, as well as therapeutic targets.

## Genomic Characterization Revealed Novel Molecular Subtypes in PCa

In recent years, several large-scale genomic studies have been performed with the aim of defining molecular subtypes in PCa that could possibly improve or complement the histopathological classification currently used in medical practice (38–45). The major genomic alterations occurring in PCa, including TMPRSS2:ERG translocations and mutations in PTEN, P53, CHD1, and SPOP, have been defined (42, 43, 46). Moreover, gene expression profiles associated to these genomic subtypes have advanced our knowledge of the molecular mechanisms driving these tumors, but our understanding of PCa progression remains limited. Although both microarray and RNA-sequencing techniques provide high degree of information with broad coverage, the correlation between mRNA and protein expression is only partial (47–49), therefore our understanding of PCa proteome remains incomplete.

## Proteomic Profiling of PCa Tissues

The utilization of several proteomic techniques in PCa profiling has been recently reviewed (29, 30). Bottom-up approaches, that is, protein identification from the analysis of their peptide components, has so far resulted in better proteome coverage. Tryptic digestion followed by peptide fractionation, peptide separation through liquid chromatography, and on-line electrospray ionization (50) coupled to a MS/MS orbitrap-based analyzers (51) is the most extended choice for in-depth proteomic analysis^1^ (52) (Figure 1). Improvements in sample preparation, the sensitivity of mass analyzers and computational developments now allows to quantify what is considered to be close to a full proteome (over 10,000 proteins expressed per cell) (48, 53, 54). This depth of coverage is state-of-the-art today but will become standard in the near future. Alternative approaches such as the SWATH-MS methodology developed by Aebersold’s laboratory, based on data-independent acquisition, can provide large coverage with high degree of reproducibility (55). However, this methodology has rarely been applied to PCa research so far (4).

Of all the proteomic profiles performed on PCa tumors, two studies stand out for the extensive coverage achieved (6, 13). Drake et al. reported the phosphoproteomic profile of 16 metastatic castration-resistant samples obtained from 13 patients, together with 6 prostate localized tumors and 5 benign prostate tissue obtained from autopsy programs (13). They identify and quantify over 8,000 phospho-peptides using label-free mass spectrometry. Integration of these data with other genome-wide approaches allowed them to identify kinase-regulated signaling pathways activated in castration-resistant PCa compared to untreated tumors including DNA repair, PI3K–AKT–MTOR, and cell cycle progression related processes. The use of drugs targeting some of the kinases involved in the pathways that are active in metastatic PCa are currently being tested in clinical trials^2^ (clinical trial IDs: NCT02407054 and NCT02091531).

While Drake and coworkers used frozen sample for their studies, mass spectrometry quality protein extracts can also be obtained from formalin-fixed paraffin-embedded (FFPE) tissues (6, 31–33). Due to the reduce maintenance cost and long-lasting preservation, FFPE tissue archives are the preferred option for long-term tumor storage. The use of FFPE samples opens the possibility of retrospective proteomic analysis of samples from clinical trials where long-term clinical follow-up of patients is available. This is especially important when investigating diseases such as PCa, with a disease course that sometimes expands over 25 years.

Recently, we analyzed the proteome of 36 samples obtained from FFPE preserved radical prostatectomy specimens. Twenty-eight corresponded to tumors with different histological patterns (Gleason score) and eight to neighboring non-malignant tissue (6). An average of 5,580 (±515) proteins were identified for a total sum of over 9,000. Accurate relative quantification was achieved by the use of extracts from SILAC (Stable-Isotope Labeling by Amino acids in Cell culture)-labeled prostate cell lines as spike-in standard for each sample (56). In addition to earlier described changes in the early progression of prostate tumors (increased expression of androgen receptor regulated proteins, reduced levels of proteins involved in cell adhesion, etc.), we showed that prostate localized tumors exhibit increased expression of mitochondrial proteins, which come accompanied by increased mitochondrial activity. This seems to be a particularity of prostate tumors compared to other types of cancer that commonly depend on glycolytic pathways for energy production. The targeting of mitochondrial function with agents like Metformin or Phenformin in combined regimens with Enzalutamide (AR inhibitor) is currently being tested in clinical trials for the treatment of metastatic PCa [(57), see text footnote 2 for clinical trial IDs: NCT01620593; NCT02339168; NCT02176161; NCT02640534; NCT00881725; NCT01864096, among others]. As part of this study, by comparing PCa tumors of different Gleason grades, we identified pro-NPY as a novel prognostic biomarker in early PCa and verify its performance in two independent large cohorts of minimally treated PCa patients. The potential role of pro-NPY as an early prognostic biomarker must now be further validated using a modern cohort of PCa patients, preferable those enrolled in active surveillance programs.

These studies constitute a great leap forward toward the complete characterization of PCa proteome and have unraveled the most general proteomic features that characterize PCa progression. However, they all show a similar limitation, which concerns to the relatively small number of samples analyzed in each study. In order to validate the utility of proteomic profiling toward personalized treatment of PCa, more tissue samples need to be analyzed, including different genetic and histological subtypes, tumors subjected to different treatment options, and tumors growing in different metastatic niches (Table 1). These questions are only starting to be investigated. For instance, proteome differences between tumors bearing or not the TMPRSS2:ERG translocation have been studied (11). Despite the small number of clinical samples analyzed and limited proteome coverage, some proteins like MYO6 were found as differentially regulated between ERG+ and ERG− tumors (11). Another example of initial proteomic study tackling a relevant PCa question is the analysis of epithelial and microenvironment tissue that were micro-dissected from tumors graded with different Gleason scores (12). Despite similar limitations as the previously described study, this investigation served as a proof of principle for the existence of major differences in tumor microenvironment. The composition of the tumor microenvironment is critical in the development of PCa (58–60). Therefore, deep proteomic characterization of this compartment is warranted in order to understand its influence in PCa progression. Many aspects of PCa tissue proteome dynamics remain to be explored. The analysis of the proteome changes occurring during metastatic dissemination of PCa (primarily to bone), in combination with the effects of androgen deprivation and chemo-therapies, including neuroendocrine differentiation, will potentially contribute to identify targets for novel therapeutic approaches and contribute to patient stratification toward personalized treatments (Table 1).

## Quantitative Proteomic Profile of Biofluids from PCa Patients

Blood and urine are ideal sources for biomarker identification due to the minimally invasive procedures of acquisition and the plentiful availability. In prostatic diseases, direct evidence of prostatic alteration can be obtained from the analysis of the seminal plasma. All these fluids are composed by less complex mix of proteins compared to tissues (61–63) but present other challenges, primarily: the dynamic range of the proteome; the variation in composition related to storage time; the intra- and inter patient variability and the relationship between the fluid components and the tumor.

In blood, the dynamic range, that is, the difference in concentration among the different proteins, spans for over 10 orders of magnitude (64). Highly abundant proteins such as albumins and globulins account from most of the serum protein content (64). Depletion of these proteins to enrich for low abundant ones is a common pre-requisite during sample preparation (35) although novel methodologies have been recently proposed to identify and quantify hundreds of plasma proteins using direct measurements (34).

Measurement of Prostate-Specific Antigen (PSA) blood levels is a routine tool for PCa diagnosis and screening (65). However, PSA levels are not informative of cancer aggressiveness, leading to overdiagnose of PCa patients with asymptomatic disease (66) and cannot discriminate between PCa and other prostate pathologies (67). Finding serum biomarkers that precisely identify PCa and show correlation with tumor aggressiveness have been investigated using mass-spectrometry-based approaches (30). Additionally, identification of prognostic biomarkers or indicators of treatment response has also been tested proteomic profiling of PCa patients’ blood (29, 30). From those studies, the identification of the pigment epithelium-derived factor (PEDF) stands out as common serum marker with altered expression during PCa progression (19–21). PEDF seems to promote macrophage recruitment to the tumors and to inhibit the expression of metalloproteases involved in PCa cells invasion (68–70), supporting a role as tumors suppressor in PCa. Therefore, the potential of PEDF as diagnostic and prognostic biomarker for PCa should be validated in larger cohorts.

Other approaches aiming at evaluating tumor characteristics through non-invasive protocols is to purify tumor-derived components from blood. This is the case of exosomes and circulating tumor cells (CTCs). Exosomes are small vesicles of 50–100 nm in diameter secreted from most tissues including the prostate (71–73). Proteomic characterization of these microvesicles has the advantage of being directly produced from the prostatic tissue both normal and cancerous, and being composed by a relatively low complex proteome, which make them potentially interesting as a source of blood biomarkers. However, isolation and enrichment of exosomes is a time-consuming process that requires larger amounts of initial material due to the relative low number of these vesicles presents in the blood (27, 74). Moreover, most methods applied today for exosome isolation from blood are based on centrifugation protocols with limited specificity for PCa cell derived vesicles (27). This limited specificity can be ignored when analyzing genetic changes specifics to PCa such as the presence of AR receptor variants, whose detection on exosomes from PCa patient’s blood can predict resistance to hormonal therapy (27). However, for proteomic profiling more specific methodologies of PCa cells derived exosome isolation would be required.

Circulating tumor cells analysis constitutes a direct source of information regarding tumor genetics. Proteomic characterization of CTCs could additionally reveal signaling pathways activated in the tumors and be used to direct personalized treatments. This is particularly relevant during the metastatic onset of the disease due to the difficulties and ethical concerns related to the collection of metastatic biopsies. However, proteomic profiling of CTCs remains extraordinarily challenging due to the limited amount available per sample. Development of single-cell mass spectrometry protocols (75) would be critical for the future proteome profile of this type of clinical material.

Urine is a plentiful source of biomarkers. In prostatic diseases, direct evidence of the prostate secretome can be obtained from urine samples, especially when prostatic massage is performed before the sample collection. Thus, clinical tests based on the measurement of the non-coding RNA PCA3 levels in urine, have been approved by the FDA to evaluate the necessity of prostate re-biopsy in men with previously negative biopsy (76–78). Similar to the analysis of blood proteome, challenges regarding wide dynamic range, and inter- and intra- individual heterogeneity apply to proteomic studies performed on urinary samples (36). In an attempt to identify novel diagnostic and prognostic biomarker for PCa, several mass spectrometry-based proteomic profiles have been performed either by direct measurements or after exosomes isolation [reviewed in Ref (79).]. Especially relevant is the identification of CD14 as urine marker to discriminate between benign prostatic hyperplasia (BPH) and PCa with high sensitivity and specificity (16). A similar attempt to discriminate between BPH and PCa using urine proteins found β2M, PGA3, and MUC3, whose levels, in combination with PSA concentration in blood, achieved a better predictive accuracy that PSA alone with a receiving operating characteristic (ROC), area under the curve (AUC) of 0.812 (17). Additionally, altered expression in urine of the proteins serotransferrin (TF), haptoglobin (HP), and AMBP was retrospectively found in men diagnosed or not with PCa (18), with an ROC AUC of 0.848. Finally, Li and coworkers described that lower concentration of peptides from osteopontin (SPP1) and prothrombin (F2) in PCa that in BPH (14). Unfortunately, despite being similar approaches, the results of the different studies do not overlap and, therefore, the clinical utility of any of these biomarkers would require independent replication in larger cohorts of PCa patients.

Non-invasive biomarkers of disease progression would help to monitor patients managed with active surveillance programs. These patients are periodically monitored for changes in blood PSA concentration and other signs of disease progression (80, 81). After proteomic profiling of urinary extracellular vesicles using mass spectrometry, Fujita et al. identified FABP5 protein differentially expressed among tumors with different histological features (25). All these findings require further validation but serve as a proof of principle that despite the common sample size limitation, deep proteomic profile of urinary samples has the potential to identify novel PCa diagnostic and prognostic biomarkers.

In addition to blood and urine, analysis of seminal fluid is particularly interesting to prostate diseases as it may provide direct evidence of alterations in the prostate gland, including the development of PCa. Early studies by Drake and coworkers identified 916 proteins from prostatic fluids obtained from PCa patients after prostatic massage. Identified proteins were enriched for those of prostatic origin, therefore, validating their approach (26). Then, they applied this methodology to discriminate between patients bearing organ-confined or extracapsular prostate tumors and identified a series of proteins and validated 3 proteins (MME, PARK7, and TIMP1) as differentially expressed between both conditions using a selective reaction monitoring (SRM)-MS targeted approach (23). Proteomics on seminal fluid has also been used to try to discriminate between indolent and advance PCa. Thus, Neuhaus et al. proposed a signature of peptides isolated from seminal fluid able to retrospectively identify patients with advance and localize prostate tumors histologically scored with similar Gleason grade (24). As for most of these studies, replication of the results in larger cohorts of patients is needed in order to conclude about the utility of the individual markers.

## Future Perspectives

Clinical management of PCa can benefit from the fast-paced development within the mass spectrometry field (Table 1). Understanding PCa natural progression and identifying signaling pathways that turn deregulated during this evolution is critical to find novel and more personalized treatments. Proteomic characterization of metastatic PCa has the potential to increase our understanding of the lethal stage of the disease. The identification of prognostic biomarkers that can predict tumor aggressiveness or response to therapies at early stages are needed to limit the unnecessary treatment of patients bearing low-risk tumors, while offering the most adequate treatment to each patient at the earliest possible time during disease progression.

Current mass spectrometry-based proteomic techniques allow determination of numerous posttranslational modifications, including phosphorylation, glycosylation, and ubiquitination (82–84). However, application of these methodologies to clinical PCa samples is scarce. Deep profiling of these protein modifications will provide new insights regarding the cellular pathways activated during disease progression and would help to develop novel therapeutic approaches and identification of novel diagnostic and prognostic biomarker in PCa. Moreover, the use of targeted proteomic technologies, such as SRM and MRM, which can provide accurate and reproducible measurement of biomarkers of interest (85–89), may be critical for the clinical use of the identified biomarkers. An example is the development of such targeted approach to identify different isoforms of SPOP, a commonly mutated gene in PCa (90). In addition, improved protocols and technical developments are still necessary to allow for the proteomic characterization of, for instance, CTCs.

Finally, integration of mass spectrometry-based proteomic profiling with other state-of-the-art technologies, especially with next-generation sequencing, will provide specific information of the signaling pathways and processes activated in a given patient with its unique tumor genetic background, helping to elucidate the functional consequences of somatic mutations, define driver proteins, and identify therapeutic targets. Integration of these preoteogenomic approaches (91) has already been applied to other cancer types (49, 92) and will be critical to achieve full personalized management in PCa.

## Author Contributions

Collection of data and drafting the manuscript: AF-M and DI-G.

## Conflict of Interest Statement

The authors declare that the research was conducted in the absence of any commercial or financial relationships that could be construed as a potential conflict of interest.

## Footnotes

^1^http://www.proteomexchange.org.

^2^https://clinicaltrials.gov.
